# Glycation Interferes with the Expression of Sialyltransferases in Meningiomas

**DOI:** 10.3390/cells10123298

**Published:** 2021-11-25

**Authors:** Philipp Selke, Kaya Bork, Tao Zhang, Manfred Wuhrer, Christian Strauss, Rüdiger Horstkorte, Maximilian Scheer

**Affiliations:** 1 Medical Faculty, Institute for Physiological Chemistry, Martin-Luther-University Halle-Wittenberg, 06114 Halle (Saale), Germany; kaya.bork@medizin.uni-halle.de (K.B.); Ruediger.horstkorte@medizin.uni-halle.de (R.H.); maximilian.scheer@uk-halle.de (M.S.); 2 Center for Proteomics and Metabolomics, Leiden University Medical Center, 2333 ZA Leiden, The Netherlands; t.zhang@lumc.nl (T.Z.); m.wuhrer@lumc.nl (M.W.); 3 Department of Neurosurgery, University Hospital Halle, 06120 Halle (Saale), Germany; Christian.strauss@uk-halle.de

**Keywords:** intracranial tumor, methylglyoxal, MGO, sialylation, tumorigenesis, posttranslational modification

## Abstract

Meningiomas are the most common non-malignant intracranial tumors and prefer, like most tumors, anaerobic glycolysis for energy production (Warburg effect). This anaerobic glycolysis leads to an increased synthesis of the metabolite methylglyoxal (MGO) or glyoxal (GO), which is known to react with amino groups of proteins. This reaction is called glycation, thereby building advanced glycation end products (AGEs). In this study, we investigated the influence of glycation on sialylation in two meningioma cell lines, representing the WHO grade I (BEN-MEN-1) and the WHO grade III (IOMM-Lee). In the benign meningioma cell line, glycation led to differences in expression of sialyltransferases (*ST3GAL1/2/3/5/6, ST6GAL1/2, ST6GALNAC2/6*, and *ST8SIA1/2*), which are known to play a role in tumor progression. We could show that glycation of BEN-MEN-1 cells led to decreased expression of ST3Gal5. This resulted in decreased synthesis of the ganglioside GM3, the product of ST3Gal5. In the malignant meningioma cell line, we observed changes in expression of sialyltransferases (*ST3GAL1/2/3, ST6GALNAC5*, and *ST8SIA1*) after glycation, which correlates with less aggressive behavior.

## 1. Introduction

Meningiomas arise from the arachnoid and are the most common non-malignant intracranial tumor [1,2,3,4,5]. They are classified according to WHO (World Health Organization) in grades I, II, and III. The benign grade I represents the most frequent subtype (>80%), and has a low risk of recurrence and slow growth [5,6]. As opposed to benign meningioma, grade III meningiomas (anaplastic, rhabdoid, and papillary subtype) are rare (1–3%) and little is known about factors that influence their survival and malignity. The present surgical, medicinal, and radiotherapeutic treatments are not adequate to manage the morbidity and mortality in this subtype [7,8,9,10].

Like many other tumors, meningiomas use glucose as a primary energy source (Warburg effect), which is considered as one of the “hallmarks of cancer” [11,12,13].

During glycolysis, up to 0.4% of the glucose is converted into methylglyoxal (MGO). MGO is a typical side product of glyceraldehyde-3-phosphate, which is generated by the aldolase reaction from fructose-1,6-bisphosphate. Please note that MGO is more than 20,000 times more reactive than glucose [11]. Previous studies showed that MGO concentrations are elevated in diabetic and or aged individuals [14]. Many studies suggest that diabetes is linked to an increased risk of cancer [15,16]. In line with this, there is a correlation between serum glucose levels and meningioma risk [17,18]. However, there are contrary data suggesting a positive [19,20] or inverse [21] correlation between diabetes and serum glucose levels and the risk of meningioma. For example, patients with type 2 diabetes have a decreased survival after surgical resection of a WHO grade I meningioma [22].

The dicarbonyl MGO reacts primarily with proteins (through arginine, lysine, and cysteine residues) or to a small extent also with DNA and lipids. This non-enzymatic reaction between the carbonyl groups of dicarbonyls (i.e., MGO) or monosaccharides (i.e., glucose) and the amino groups of proteins is called glycation [23,24]. Another important glycating agent is glyoxal (GO), which is formed by degradation of glucose or autoxidation of glycoaldehyde to glyoxal [25]. Glycation is much stronger with dicarbonyls than with monosaccharides [26]. The end products of this reaction are called advanced glycation end products (AGEs) [27,28,29]. Recently, we demonstrated that glycation through MGO led to an increased invasive behavior in benign meningioma cells [30]. Several other studies propose MGO as a tumor-promoting agent [31,32].

Another common posttranslational modification is glycosylation. In contrast to glycation, glycosylation is an enzymatic addition of carbohydrates, glycans to a non-carbohydrate-structure, commonly a lipid or protein in the endoplasmatic reticulum (ER)/Golgi. Sialylation is of deep interest and describes the addition of sialic acids (Sia) to lipids (i.e., gangliosides) or proteins (i.e., neural cell adhesion molecule (NCAM)) through sialyltransferases (ST) [33].

**Figure 1:**
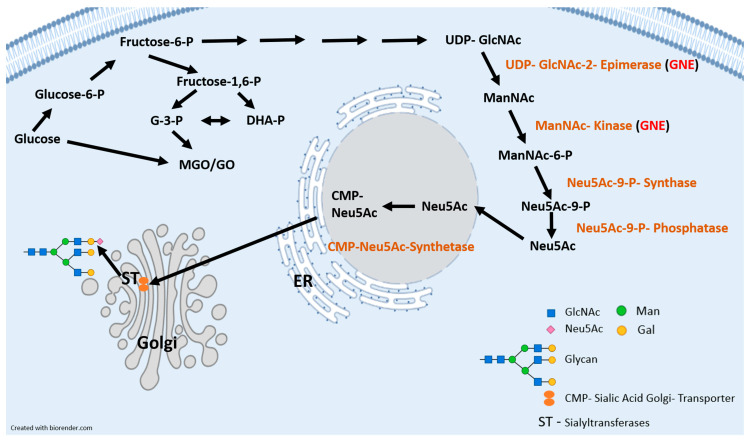
Schematic representation of the Sia biosynthesis from glucose to Sia and glycation agents (MGO/GO) and sialylation of glycoproteins (i.e., *N*-glycans, *O*-glycans or gangliosides) in the endoplasmatic reticulum and Golgi. G-3-P = glyceraldehyde-3-phosphate; DHA-P = Dihydroxyaceton phosphate; MGO = methylglyoxal; GO = glyoxal; GlcNAc = *N*-acetyl-glucosamine; Man = Mannose; Gal = Galactose; Neu5Ac = *N*-acetyl-neuraminic acid; GNE = UDP-*N*-acetyl glucosamine 2-epimerase/*N*-acetyl mannosamine kinase; ER = Endoplasmatic reticulum.

*N*-acetyl neuraminic acid (Neu_5_Ac) represents the major Sia of mammals. It is synthesized from UDP-*N*-acetyl glucosamine (UDP-GlcNAc) in the cytosol [34]. The key enzyme of the Sia biosynthesis is the bifunctional UDP-*N*-acetyl glucosamine 2-epimerase/*N*-acetyl mannosamine kinase (GNE) [35]. Sialylation is taking place in the Golgi and is catalyzed by STs. They are 20 known STs in humans, which use CMP-activated Sia as substrate (Figure 1). These STs are subdivided into 4 families dedicated to the carbohydrate linkages they synthesize: beta-galactoside alpha 2,3-sialyltransferases (ST3Gal1-6), beta-galactoside alpha 2,6-sialyltransferases (ST6Gal1-2), *N*-acetyl galactosamine (GalNAc) alpha 2,6-sialyltransferases (ST6GalNAc1-6) and alpha 2,8-sialyltransferases (ST8Sia1-6) [36,37]. The members of the ST3Gal family transfer Sia from CMP-Sia to terminal galactose residues through 2,3 linkages, whereas the two known members of the ST6Gal family do this through 2,6 linkages. The six members of the ST6GalNAc family transfer Sia from CMP-Sia to GalNAc residues via 2,6 linkages. In addition, the ST8Sia-family transfer Sia from CMP-Sia to other terminal Sia residues by 2,8-linkages [36,37]. High blood glucose concentrations in individuals with diabetes result in a UDP-GlcNAc-dependent change to more complex *N*-glycans [38]. Especially, glycoproteins with only few *N*-glycosylation sites such as transforming growth factor β (TGFβ) or glucose transporter 4 (GLUT4) show rapid response to increasing GlcNAc concentrations causing complex glycan formation and branching [39]. Gangliosides are glycosphingolipids that contain Sias. The synthesis of gangliosides begins with ceramide (Cer) in the ER. During GM3-synthesis, Cer will be glucosylated by the glucosylceramid synthase. After this step in the cis-golgi, glucosylceramide is converted in the trans-golgi to lactosylceramide [40]. This is the substrate for GM3-synthase (ST3Gal5). It is known that GM3 plays a role during several diseases (chronic inflammation, insulin resistance or cancer) [41,42,43].

In this study, we compared the expression of STs in benign and malignant meningioma cells and found significant differences between these two. Furthermore, we investigated the role of the glycating metabolite MGO on the expression of STs in both benign and malignant meningioma cells. Thereby, we could show that glycation has a dramatic effect on the expression of STs and consequently on the GM3 expression. As a result, this could change sialylation-dependent tumor progression in meningioma.

## 2. Materials and Methods

### 2.1. Cell Culture

The human benign meningioma cell line BEN-MEN-1 was obtained from Leibniz-Institute DSMZ (Deutsche Sammlung von Mikroorganismen und Zellkulturen GmbH, Braunschweig, Germany) and the human malignant meningioma cell line IOMM-Lee (ATCC^®^ CRL-3370™) was obtained from American Type Culture Collection (ATCC, Manassas, VA, USA). Both cell lines were cultured in Dulbecco’s Modified Eagle’s Medium (DMEM) supplemented with 100 µg/mL of streptomycin, 100 U/mL of penicillin, 4 mM of glutamine, and 10% fetal bovine serum (FBS, Sigma-Aldrich, St. Louis, MO, USA) at 37 °C in a 5% CO_2_ incubator. The cell lines were split every 2–3 days with 0.1% Trypsin-EDTA (Ethylenediaminetetraacetic acid) solution for 2 min.

### 2.2. Glycation and Real-Time PCR Analysis

Cells were seeded in 12-well plates at a density of 3.95 × 10^4^/cm^2^ in DMEM with 1% FBS. After 2 h of attachment, the cells were treated with 0.3 mM MGO or GO. Controls (Ctrl) were cells (BEN-MEN-1, IOMM-Lee) without MGO or GO treatment. The cell lines were cultivated for 24 h. RNA was isolated using the Quick-RNA™ MiniPrep Kit (Zymo Research, Irvine, CA, USA) according to the manufacturer’s instructions. The quality and concentration of the RNA were analyzed using the NanoDrop 1000 Spectrophotometer (Thermo Fisher Scientific, Waltham, MA, USA). RNA (2 μg) was transcribed into cDNA using SuperScript™ II Reverse Transcriptase according to the manufacturer’s instructions. PCR reactions were performed using DreamTaq DNA polymerase (Thermo Fisher Scientific), and products were separated on a 1.5% agarose gel. The following conditions were used: initial denaturation for 2 min at 95 °C, 35 cycles (30 s at 95 °C, 30 s at 55 °C, 30 s at 72 °C), final elongation for 5 min at 72 °C. We use for all sialyltransferases the same primer pairs which were used in a previous study [44].

The sialyltransferase expression of untreated meningioma cell lines (BEN-MEN-1; IOMM- Lee) and after 24 h of glycation with 0.3 mM MGO were measured via quantitative real-time PCR (qPCR) using the iQ™ 5 Multicolor Real-Time PCR Detection System (Biorad, Hercules, CA, USA) and qPCR GreenMaster (Jena Bioscience, Jena, Germany) with the same primer pairs used for normal PCR. The following conditions were used for qPCR: initial denaturation for 1:30 min at 95 °C, 40 cycles (10 s at 95 °C, 10 s at 62 °C, 25 s at 72 °C), final elongation for 1 min at 72 °C, followed by a melting curve analysis. The expression level of sialyltransferases in control and glycated cell lines was determined relative to GAPDH (165 bp; fw: GGAGCGAGATCCCTCCAAA; rv: ATGACGAACATGGGGGCATC), calculated as ΔCT. The control was relatively computed to glycated cell line (2^−ΔΔCT^). All reactions were performed in triplicate.

### 2.3. Cultivation of BEN-MEN-1 Cells and Preparation of GSL-Glycan Alditols Released from BEN-MEN-1 Cells

Extraction of GSLs and preparation of GSL-glycan alditols from cells were performed in triplicate as previously described [45]. The cells were cultivated until 80% of confluence and followed by 24 h treatment with and without 0.3 mM MGO. Shortly, 2 × 10^6^ cells were harvested, washed and resuspended with 200 μL of water. The cell samples were lysed by vortexing and sonication for 30 min. In this step, 2.5 µL of 0.5 µM ganglioside GT1b in ethanol were added as a spiked internal standard to monitor sample preparation and to normalize roughly absolute quantification. Chloroform (550 μL) was added to the samples followed by 15 min sonication. Methanol (350 μL) was added to the cell pellets and incubated for 4 h with shaking at room temperature. The upper phase containing GSLs was collected after centrifugation at 2700× *g* for 20 min. Then, 400 μL of chloroform/methanol (2:1, *v*/*v*) was added, followed by adding 400 μL of methanol/water (1:1, *v*/*v*). After sonication and centrifugation, the upper phase was collected and pooled to the previous sample. The process of adding methanol/water (1:1, *v*/*v*), sonication, centrifugation and removing the upper phase was repeated another two times. In each replicate, the upper phase was collected and replaced by the same volume of methanol/water (1:1, *v*/*v*). The combined upper phases were dried under vacuum in an Eppendorf Concentrator 5301 (Eppendorf, Hamburg, Germany) at 30 °C.

Before the purification of the GSLs using reverse-phase (RP) SPE, the samples were dissolved in 100 μL methanol followed by the addition of 100 μL water. TC18-RP-cartridges were prewashed with 2 mL of chloroform/methanol (2:1, *v*/*v*), 2 mL of methanol followed by equilibration with 2 mL methanol/water (1:1, *v*/*v*). The extracted GSLs were loaded to the cartridge and washed with 2 mL methanol/water (1:1, *v*/*v*). The GSLs were eluted from the column with 2 mL methanol and 2 mL chloroform/methanol (2:1, *v*/*v*). The samples were dried under vacuum in an Eppendorf Concentrator at 30 °C.

To release the glycans from the GSLs, a mixture of EGCase I (12 mU, 2 μL), EGCase I buffer (4 μL) and water (34 μL) (pH 5.2) was added to each sample and incubated for 36 h at 37 °C. The released glycans were collected and loaded on TC18-RP-cartridges, which had been preconditioned with 2 mL of methanol and 2 mL of water. The samples were washed with 200 μL of water and residual glycans were loaded to the cartridge. Then, 500 μL of water were added to the cartridge to wash the glycans from the column. The flow-through and wash fractions were pooled and dried in an Eppendorf Concentrator at 30 °C.

The reduction was carried out with slight modifications following the same procedure as described in previous work [45,46]. In brief, GSL-glycans were reduced to alditols in 20 μL of sodium borohydride (500 mM) in potassium hydroxide (50 mM) for 2 h at 50 °C. Subsequently, 2 μL of glacial acetic acid were added to acidify the solution and quench the reaction. The desalting of GSL-glycans was performed as previously described. Glycan alditols were eluted with 50 μL of water twice. The combined flow-through and eluate were pooled and dried under vacuum in an Eppendorf Concentrator at 30 °C. The carbon SPE clean-up was performed and the purified glycan alditols were re-suspended in 20 μL of water prior to Porous Graphitized Carbon (PGC) nano-Liquid Chromatography (LC)-Electro Spray Ionization (ESI)-Mass Spectrometry (MS)/MS/MS analysis.

### 2.4. Analysis of GSL-Glycan Alditols Using PGC Nano-LC-ESI-MS/MS

The analysis of glycan alditols was performed using PGC nano-LC-ESI-MS/MS following a method described previously [45,46]. Measurements were performed on an Ultimate 3000 Ultra-High-Performance Liquid Chromatography (UHPLC) system (Thermo Fisher Scientific) equipped with a home-packed PGC trap column (5 μm Hypercarb, 320 μm × 30 mm) and a home-packed PGC nano-column (3 μm Hypercarb 100 μm × 150 mm) coupled to an amaZon ETD speed ion trap (Bruker, Bremen, Germany). Mobile phase A consisted of 10 mM ABC, while mobile phase B was 60% (*v*/*v*) acetonitrile/10 mM ABC. The trap column was packed with 5 μm particle size PGC stationary phase from Hypercarb PGC analytical column (size 100 × 4.6 mm, 5 μm particle size, Thermo), while the PGC nano-column was packed with 3 μm particle size PGC stationary phase from Hypercarb PGC analytical column (size 30 × 4.6 mm, 3 μm particle size, Thermo).

To analyze glycans, 2 μL injections were performed and trapping was achieved on the trap column using a 6 μL/min loading flow in 1% solvent B for 5 min. Separation was achieved with a linear gradient from 1% to 50% solvent B over 73 min, applied followed by a 10 min wash step using 95% of B at a 0.6 μL/min flow rate. The column was held at a constant temperature of 35 °C.

Ionization was achieved using the nanoBooster source (Bruker) with a capillary voltage of 1000 V applied and a dry gas temperature of 280 °C at 3 L/min and isopropanol enriched nitrogen at 3 psi. MS spectra were acquired within an *m/z* range of 340–1850 in enhanced mode using negative ion mode, smart parameter setting was set to *m/z* 900. MS/MS spectra were recorded using the top 3 highest intensity peaks.

Structures of detected glycans were studied by MS/MS in negative mode. Glycan structures were assigned based on the known MS/MS fragmentation patterns in negative-ion mode [47,48], elution order, and general glycobiological knowledge, with the help of Glycoworkbench [49] and Glycomod [50] software. To get an estimate of the glycan amount per cell, glycan intensity was normalized to the intensity of the internal standard GT1b. Then, assuming complete release of glycans and similar response factors between released glycan and GT1b standard, the number of glycans per cell was estimated.

Structures are depicted according to the Consortium of Functional Glycomics (CFG). Blue square is *N*-acetylglucosamine; yellow square is *N*-acetylgalactosamine; blue circle is glucose; yellow circle is galactose; red triangle is fucose; purple diamond is *N*-acetylneuraminic acid, grey diamond is *N*-glycolylneuraminic acid.

### 2.5. Statistical Analysis

All analyses and visualizations were performed using OriginPro 2019 software (OriginLab Corporation, Northampton, MA, USA). Paired Student *t*-test against the control group, both cell lines of a theoretical value of 1 (due to data normalization), were executed. Figures show the average mean with standard deviation (SD) and levels of significance are represented within the figures.

## 3. Results

### 3.1. Expression of Sialyltransferases in Meningioma Cell Lines

**Figure 2:**
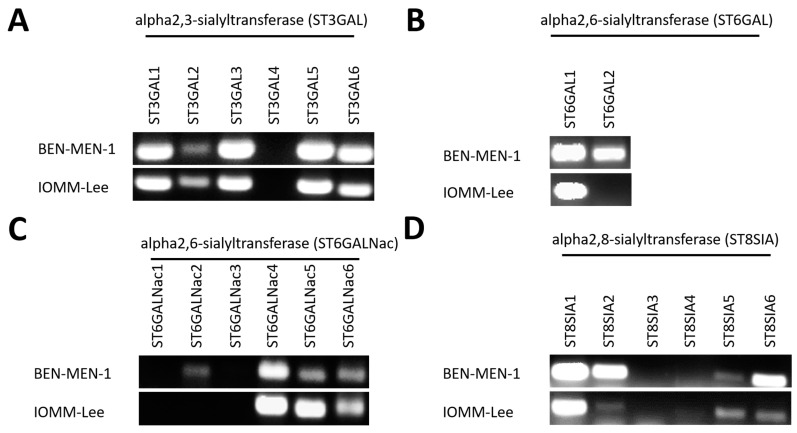
Expression of 20 Sialyltransferases (agarose gel) in BEN-MEN-1 and IOMM-Lee. (**A**): Expression of *ST3GAL1–6*. (**B**)**:** Expression of *ST6GAL1–2*. (**C**)**:** Expression of *ST6GALNAC1–6*. (**D**)**:** Expression of *ST8SIA1–6*.

Since there is evidence that sialyltransferases have an impact on tumorigenesis, we analyzed benign (BEN-MEN-1) and malignant (IOMM-Lee) meningioma cell lines regarding differences in expression of sialyltransferases (Figure 2, Table 1). Figure 2A shows the expression of *ST3GAL1–6* in BEN-MEN-1 and IOMM-Lee. *ST3GAL1–ST3GAL3* and ST3GAL5–ST3GAL6 were detected in both cell lines. The band intensity of ST3GAL2 was higher in the malignant cell line compared with the benign cell line, whereas the *ST3GAL3, ST3GAL5–6* band intensities were higher in BEN-MEN-1 compared to the malignant cell line. Agarose gel of *ST6GAL1–2* is shown in Figure 2B for both cell lines. *ST6GAL2* was only expressed in the benign cell line. In contrast, no differences could be found in terms of band intensity of *ST6GAL1* in both cell lines. The expression of *ST6GALNAC1–6* for both cell lines is shown in Figure 2C. In contrast to IOMM-Lee, a weak band in *ST6GALNAC2* was detectable in BEN-MEN-1. Expression of *ST6GALNAC4–6* was detectable in both cell lines. The band intensities of *ST6GALNAC5* and *ST6GALNAC6* were higher in IOMM-Lee compared to BEN-MEN-1. The expression of *ST8SIA1–6* in both cell lines is shown in Figure 2D. For *ST8SIA1–2* and *ST8SIA5–6*, the expression has been detected in both meningioma cell lines. In BEN-MEN-1, we observed a higher expression of *ST8SIA2* and *ST8SIA6* compared to the malignant cell line. Again, the band intensity of *ST8SIA5* was stronger in IOMM-Lee compared to the benign cell line.

### 3.2. Sialyltransferases Are More Affected by MGO in Benign Cell Line

**Figure 3:**
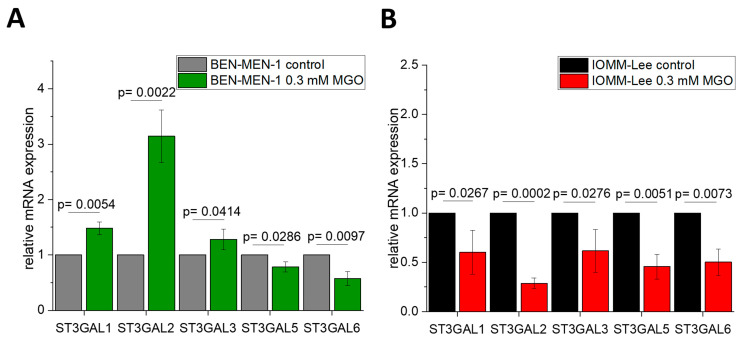
Relative mRNA expression of *ST3GAL1–6* in BEN-MEN-1 (**A**) and IOMM-Lee (**B**). (**A**): Normalized control (grey) and mRNA expression after 24 h treatment with 0.3 mM MGO (green). (**B**): Normalized control (black) and mRNA expression after 24 h treatment with 0.3 mM MGO (red). Statistical analysis was performed using *t*-test and error bars represent SD (n = 4; *ST3GAL1*: *p* = 0.0054 (**A**), *p* = 0.0267 (**B**); *ST3GAL2*: *p* = 0.0022 (**A**), *p* = 0.0002 (**B**); *ST3GAL3*: *p* = 0.0414 (**A**), *p* = 0.0276 (**B**); *ST3GAL5*: *p* = 0.0286 (**A**), *p* = 0.0051 (**B**); *ST3GAL6*: *p* = 0.0097 (**A**), *p* = 0.0073 (**B**)).

Since the expression of sialyltransferases is different in the benign and malignant meningioma cell lines, we quantified the sialyltransferase mRNA expression level after 24 h of glycation of the cells to verify the influence of glycation on sialylation. Figure 3 displays the different mRNA expressions of *ST3GAL1–6* in BEN-MEN-1 (Figure 3A) and IOMM-Lee (Figure 3B). Glycation led to changes in ST expression. In the benign cell line, we observed an increased overall expression, whereas we noticed a decreased overall expression of STs in the malignant cell line. *ST3GAL1* (1.4812 ± 0.115 fold change), *ST3GAL2* (3.143 ± 0.476 fold change), and *ST3GAL3* (1.28 ± 0.189 fold change) expression were increased in contrast to non-glycated cells in BEN-MEN-1. Furthermore, the relative expression of *ST3GAL5* (0.7863 ± 0.0933 fold change) and *ST3GAL6* (0.572 ± 0.126 fold change) were decreased in contrast to non-glycated cells. The relative expression of *ST3GAL1* (0.601 ± 0.223 fold change), *ST3GAL2* (0.288 ± 0.0535 fold change), *ST3GAL3* (0.6175 ± 0.217 fold change), *ST3GAL5* (0.4561 ± 0.1271 fold change), *ST3GAL6* (0.502 ± 0.1325 fold change) were decreased in contrast to non-glycated cells in the malignant cell line.

**Figure 4:**
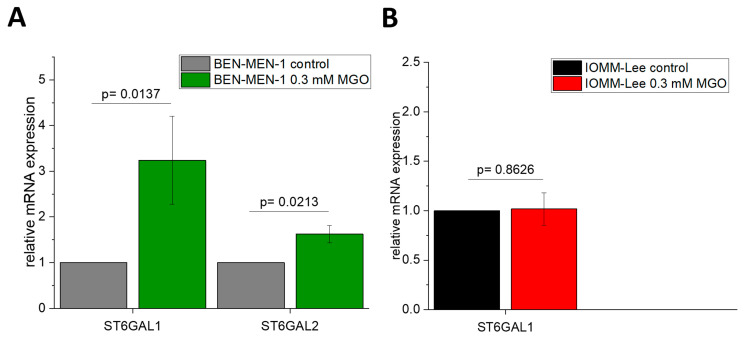
Relative mRNA expression of *ST6GAL1–2* in BEN-MEN-1 (**A**) and IOMM- Lee (**B**). (**A**): Normalized control (grey) and mRNA expression after 24 h treatment with 0.3 mM MGO (green). (**B**): Normalized control (black) and the mRNA expression after 24 h treatment with 0.3 mM MGO (red). Statistical analysis was performed using *t*-test and error bars represent SD (*n* = 4; *ST6GAL1*: *p* = 0.0274 (**A**), *p* = 0.863 (**B**); *ST6GAL2*: *p* = 0.0213 (**A**)).

Figure 4 shows the expression of the *ST6GAL*-family in BEN-MEN-1 (Figure 4A) and IOMM-Lee (Figure 4B). Glycation led to opposing changes in the expression of this sialyltransferase. We observed a higher expression in the benign cell line after treatment with MGO (3.2402 ± 0.962 fold change), whereas no changes could be measured in the glycated malignant cell line (1.018 ± 0.164 fold change). The expression of *ST6GAL2* was only detected in BEN-MEN-1 and increased after treatment with MGO (1.624 ± 0.188 fold change).

**Figure 5:**
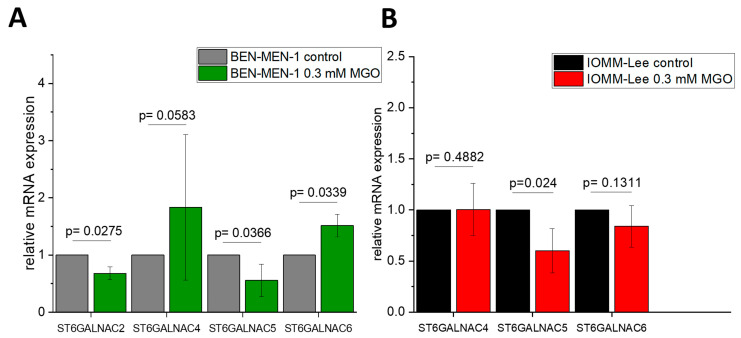
Relative mRNA expression of *ST6GALNAC1–6* in BEN-MEN-1 (**A**) and IOMM- Lee (**B**). (**A**): Normalized control (grey) and mRNA expression after 24 h treatment with 0.3 mM MGO (green). (**B**): Normalized control (black) and mRNA expression after 24 h treatment with 0.3 mM MGO (**red**). Statistical analysis was performed using *t*-test and error bars represent SD (n = 4; *ST6GALNAC2*: *p* = 0.0275 (**A**); *ST6GALNAC4*: *p* = 0.0583 (**A**), *p* = 0.4882 (**B**); *ST6GALNAC5*: *p* = 0.0366 (**A**), *p* = 0.024 (**B**); *ST6GALNAC6*: *p* = 0.0339 (**A**), *p* = 0.1311 (**B**)).

Moreover, the mRNA expression of *ST6GALNAC1–6* in BEN-MEN-1 (Figure 5A) and IOMM-Lee (Figure 5B) is also differently altered after glycation. *ST6GALNAC2* expression decreased after glycation in contrast to the untreated benign cell line (0.6807 ± 0.1106 fold change). *ST6GALNAC4* expression is not affected in BEN-MEN-1 (2.556 ± 1.232 fold change) and IOMM-Lee (1.005 ± 0.2552 fold change), but glycation decreased the mRNA expression of *ST6GALNAC5* in both cell lines (0.5575 ± 0.283; 0.5991 ± 0.2174). Glycation led to a higher expression of *ST6GALNAC6* in the benign cell line (1.5141 ± 0.1999). *ST6GALNAC6* expression is not influenced by glycation in the malignant cell line (0.839 ± 0.203).

**Figure 6:**
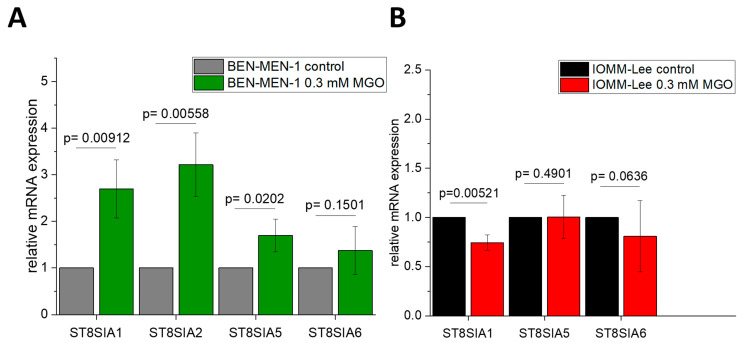
Relative mRNA expression of *ST8SIA1–6* in BEN-MEN-1 (**A**) and IOMM- Lee (**B**). (**A**): Normalized control (grey) and mRNA expression after 24 h treatment with 0.3 mM MGO (green). (**B**): Normalized control (black) and mRNA expression after 24 h treatment with 0.3 mM MGO (red). Statistical analysis was performed using *t*-test and error bars represent SD (n = 4; *ST8SIA1*: *p* = 0.00912 (**A**), *p* = 0.00521 (**B**); *ST8SIA2*: *p* = 0.00558 (**A**); *ST8SIA5*: *p* = 0.0202 (**A**), *p* = 0.4901 (**B**); *ST8SIA6*: *p* = 0.1501 (**A**), *p* = 0.0636 (**B**)).

Finally, we quantified the expression of *ST8SIA 1–6*. Glycation influenced more strongly the expression level of these sialyltransferases in BEN-MEN-1 cells compared to the malignant IOMM-Lee cell line. The expression of *ST8SIA1* was highly increased (2.696 ± 0.627 fold change) after glycation in the benign cell line (Figure 6A) and decreased (0.744 ± 0.07712 fold change) in the glycated malignant cell line (Figure 6B). The expression of *ST8SIA2* (3.2171 ± 0.6837 fold change) and *ST8SIA5* (1.696 ± 0.3475 fold change) were both increased in BEN-MEN-1 (Figure 6A). *ST8SIA5* expression was not influenced by glycation in IOMM-Lee cells. The expression of *ST8SIA6* was not influenced by glycation in both cell lines.

### 3.3. MGO-Treatment Decreases Ganglioside GM3 Expression in BEN-MEN-1

**Figure 7:**
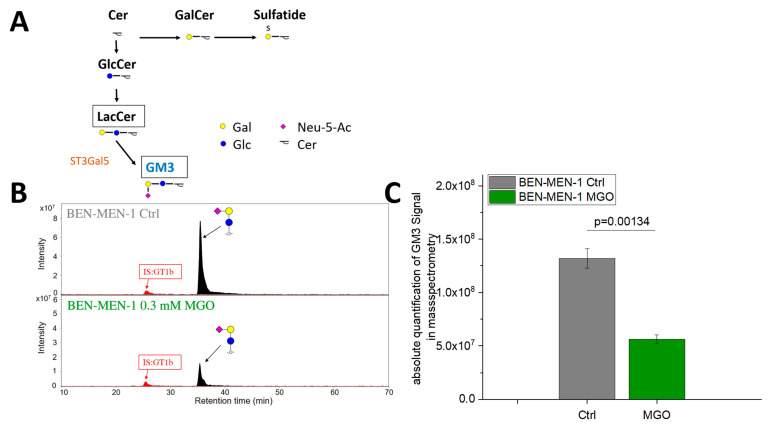
Absolute quantification of GM3 signal by PGC nano-LC-ESI-MS/MS. (**A**): Schematic representation of the GM3 biosynthesis. Cer = Ceramide; GalCer = Galactosylceramide; GlcCer = Glucosylceramide; LacCer = Lactosylceramide; GM3 = Monosialoganglioside 3. (**B**): Signal intensity of GM3 in BEN-MEN-1 Ctrl and BEN-MEN-1 0.3 mM MGO in comparison to the internal standard GT1b. (**C**): Absolute quantification of GM3 signal by PGC nano-LC-ESI-MS/MS of Ctrl (grey) and 0.3 mM MGO-treated BEN-MEN-1 (green). Statistical analysis was performed using *t*-test and error bars represent SD (n = 3; *p* = 0.00134).

To prove that MGO-induced reduction of ST3GAL5 expression has an impact on BEN-MEN-1 cells, we quantified ganglioside GM3 by PGC nano-LC-ESI-MS/MS. Figure 7A summarizes the biosynthesis of GM3. Figure 7B shows the signal intensity of GM3 before and after glycation in comparison to the internal standard of GT1b in BEN-MEN-1 cells. The absolute quantification of GM3 is shown in Figure 7C. We could show a decreased GM3 expression after glycation (*p* = 0.00314), which is in line with the decreasing expression of *ST3GAL5* (see: Figure 3A). The normalized copy numbers of GM3 per cell in the untreated BEN-MEN-1 cell line (1.32 × 10^8^ ± 9.15 × 10^6^) were decreased by 235% compared to the glycated cells (5.61 × 10^7^ ± 3.89 × 10^6^).

### 3.4. Glyoxal-Treatment Has Different Effects in ST3GAL5

**Figure 8:**
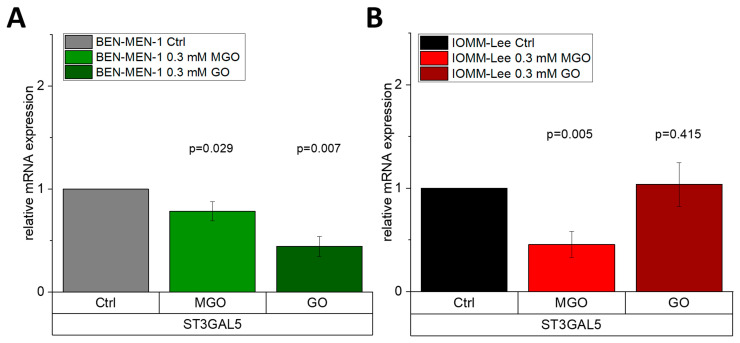
Relative mRNA expression of *ST3GAL5* in BEN-MEN-1 (**A**) and IOMM-Lee (**B**). A shows the relative expression of *ST3GAL5* in untreated (grey), 0.3 mM MGO (green)- and 0.3 mM GO (dark green)-treated BEN-MEN-1. B shows the relative expression of *ST3GAL5* in untreated (black), 0.3 mM MGO (red)- and 0.3 mM GO (dark red)-treated IOMM-Lee. Statistical analysis was performed using *t*-test and error bars represent SD (n = 4; *ST3GAL5* GO (**A**) *p* = 0.007; (**B**) *p* = 0.415).

Finally, we analyzed whether another glycation agent than MGO has the same effect on *ST3GAL5* expression as MGO. We could show by Western blot analysis that 0.3 mM glyoxal (GO) leads to glycation in both cell lines (data not shown). Using qPCR of cDNA of BEN-MEN-1 cells (Figure 8A) or IOMM-Lee (Figure 8B), which were grown for 24 h in the presence of 0.3 mM GO, we could show that GO-induced glycation had the same effect on *ST3GAL5* expression as MGO treatment in BEN-MEN-1 cells. However, GO did not alter the expression of *ST3GAL5* in malignant IOMM-Lee cells, which is in contrast to MGO. This suggests a glycation agent-specific change of *ST3GAL5* expression.

## 4. Discussion

Many studies demonstrated that sialylation has an impact on tumorigenesis [51,52,53,54,55]. Abnormal levels of different glycosyltransferases were found in different types of human cancers [56,57]. In addition, high serum levels of sialyltransferases are associated with the progression of advanced breast cancer [58].

However, little is known about the influence on glycosylation by glycation, which is increased in several cancers because of the Warburg effect [59,60]. In this study, we could show, that glycation affects sialylation by modulating ST expression, which could have an impact on different ganglioside patterns and thereby on tumor development. Most of the STs were expressed in both BEN-MEN-1 and IOMM-Lee cell lines. Glycation of both cell lines resulted in an increasing level of STs in the benign meningioma cells and decreasing level in the malignant cells.

There are many reports of changes in ST expression in cancer. Overexpression of *ST3GAL1* in ovarian cancer led to transforming growth factor (TGF)-β1-induced epithelial-mesenchymal-transition, migration, and invasion, and a knockdown resulted in the opposite [61]. Another study by Mehta et al. has revealed that *ST3GAL2* and *ST6GAL1* were significantly upregulated in tumors with positive perineural invasion status [62], which we observed in the glycated benign cell line. *ST3GAL3* was increased in the glycated BEN-MEN-1 cell line but decreased in glycated IOMM-Lee cells. Expression of *ST3GAL3* is important for the regulation of biosynthesis of brain disialoganglioside (GD)1a and trisialoganglioside (GT)1b [57]. In several studies, the altered expression of *ST3GAL3* has an impact on cell adhesion and invasion. Glycation of meningioma cell lines resulted in decreased *ST3GAL5* expression in both cell lines. This sialyltransferase is also known as monosialoganglioside (GM3) synthase [63] and suppresses the epidermal growth factor receptor (EGFR) phosphorylation, which influences the cell proliferation [64] and the cellular resistance to oxidative stress and radiation therapy through upregulation of extracellular signal-regulated kinases (ERK) [42]. The total amount of GM3 was decreased in BEN-MEN-1 cells after glycation. Yamashita and colleagues reported that GM3 synthase knockout mice displayed enhanced ligand-induced insulin receptor phosphorylation. Furthermore, they could show that an increased sensitivity in glucose and insulin tolerance consequently results in an elevated insulin signaling response [65]. Other studies show that decreasing expression of GM3 leads to decreased cell motility and cell adhesion through ERK phosphorylation along with Ras upregulation. This regulates migration through mitogen-activated protein kinase (MAPK) [41,42,66,67]. The glycating agent GO has the same effect in BEN-MEN-1 cells (downregulation of *ST3GAL5*) as MGO. However, in IOMM-Lee cells, we observed no effect after glycation with GO, which could be explained by higher glyoxalase 1 activity, which degrades dicarbonyls and has been described in many studies on cancer and glycation [68,69,70]. The expression of *ST3GAL6* was reduced in both glycated meningioma cell lines, which is known to play a key role in the generation of functional Sialyl Lewis X [71]. Decreasing levels of *ST3GAL6* can lead to decreasing migration and invasion in 5637 and J82 UBC cells as well as decreasing adhesion and migration in multiple myeloma cells [72,73].

Increased *ST6GAL1* expression, as we have shown in glycated BEN-MEN-1 cells, was also found in lung, colon, glioma, prostate, cervical, and breast cancer tissues [74,75,76,77,78,79,80]. The downregulation of *ST6GAL1* decreased metalloproteinases (MMPs) expression and suppressed invasive potential of A549 and H1299 cells in vitro [79], whereas bladder cancer has *ST6GAL1* upregulation, a tumor-suppressive role [81]. The upregulation of *ST6GAL2* was found in different types of cancer and was associated with breast cancer with higher expression of intracellular adhesion molecule (ICAM)-1, vascular adhesion molecule (VCAM)-1, CD24, MMP2, MMP9 and C-X-C motif chemokine receptor (CXCR)4 [82,83].

*ST6GALNAC2* is known as a metastasis suppressor in breast cancer and a low expression of it, as we observed in glycated BEN-MEN-1 cells, is associated with a bad prognosis [84,85]. In Colorectal Carcinoma, Venkitachalam et al. have observed the same [86]. In contrast, Schneider et al. could show, that a high expression of *ST6GALNAC2* correlates with metastases to the lymph system [87]. The expression level may be a prognostic marker, but it seems that the mutation of the gene is more important. In our study, *ST6GALNAC4* expression was elevated in glycated BEN-MEN-1. High expression of *ST6GALNAC4* leads to the prevention of O-glycan chain elongation [88]. In another study of Follicular Thyroid Carcinoma (FTC)-238 cells, the suppression of the *ST6GALNAC4* gene led to an inhibition of invasive behavior in vitro and in vivo [89]. The lower expression of *ST6GALNAC5* in both glycated meningioma cell lines in our study could be a sign of transformation, because it is restricted to the brain and synthesizes GD1alpha in the nervous tissues [90,91]. We have observed an increased expression of *ST6GALNAC6* in glycated BEN-MEN-1 cells. A study in colon cancer has shown that *ST6GALNAC6* is responsible for the synthesis of sialyl Lewis (a), which is a significant inductive mechanism in cancer progression [92,93].

The increased expression of *ST8SIA1* in glycated BEN-MEN-1 cells could lead to a weak prognosis for patients. In contrast, we observed decreased expression of *ST8SIA1* after glycation in IOMM-Lee cells. In melanoma brain metastases, it was shown that *ST8SIA1* (GD3 synthases) is upregulated and the GD3 expression is increased, which was associated with a bad prognosis [94]. In gliomas, malignancy increased by higher GD3 and GD2 expression [95]. In addition, *ST8SIA1* is one of the key drivers for malignancy in glioblastoma [96]. Mennel et al. reported on different expression levels of GD3 and GD2 in meningiomas, depending on the tumor origin [97]. A study for neuroblastoma and melanoma cells demonstrated that most neuroblastoma cells had a high expression of GD2 and melanoma cells had high expression of GD3 [98]. The increased expression of *ST8SIA2*, as we observed in glycated BEN-MEN-1 cells, plays a role in the invasive behavior and was significantly associated with the risk of relapse in non-small-cell lung carcinoma [99]. The sialyltransferase ST8Sia5, which is increased in glycated BEN-MEN-1 cells, is known to synthesize GD1c/GT1a/Tetrasialogangliotetraosyl-ceramide (GQ)1b from GM1b/GD1a/GT1b. The group of Schiopu et al. has identified thirty-four distinct glycosphingolipid components (one GM4, nine GM3, two GM2, two GD3, nine GM1, and six GD1) differing in their ceramide compositions [100].

The glycation of meningioma cell lines has opposite effects in benign or malignant meningioma cells. Overall, glycated BEN-MEN-1 cells express more sialyltransferases than unglycated, whereas glycation of IOMM-Lee cells leads to a downregulation of the sialyltransferase expression. These observations support our recent observations that glycation of BEN-MEN-1 cells lead to increased invasive potential [30].

## 5. Conclusions

To sum up, glycation of meningioma cell lines has cell line-specific effects. The glycated BEN-MEN-1 cell line is affected in a different expression of *ST3GAL1/2/3/5/6; ST6GAL1/2; ST6GALNAC2/6* and *ST8SIA1/2*. These STs have a direct or indirect impact on tumor progression. The decreased expression of *ST3GAL5* after glycation results in a decreasing expression of GM3 in benign meningioma cells. The expression levels of some sialyltransferases (*ST3GAL1/2/3; ST6GALNAC5* and *ST8SIA1*) of the glycated IOMM-Lee cell line were inhibited, which indicates less aggressive behavior.

## Figures and Tables

**Table 1:** Overview of sialyltransferase expressions in both meningioma cell lines.

GENE	BEN-MEN-1	IOMM-Lee
*ST3GAL1*	+++	+++
*ST3GAL2*	+	++
*ST3GAL3*	+++	+++
*ST3GAL4*	-	-
*ST3GAL5*	+++	+++
*ST3GAL6*	+++	+++
*ST6GAL1*	+++	+++
*ST6GAL2*	++	-
*ST6GALNAC1*	-	-
*ST6GALNAC2*	+	-
*ST6GALNAC3*	-	-
*ST6GALNAC4*	+++	+++
*ST6GALNAC5*	++	+++
*ST6GALNAC6*	++	++
*ST8SIA1*	+++	+++
*ST8SIA2*	+++	+
*ST8SIA3*	-	-
*ST8SIA4*	-	-
*ST8SIA5*	+	+
*ST8SIA6*	+++	+

Table 1 shows overview of sialyltransferase expression in both meningioma cell lines. The expression levels are displayed in +++ = high expression level; ++ = middle expression level; + = low expression level; - = no expression.

## Data Availability

The data presented in this study are available in this article.
